# Controlling Viral Immuno-Inflammatory Lesions by Modulating Aryl Hydrocarbon Receptor Signaling

**DOI:** 10.1371/journal.ppat.1002427

**Published:** 2011-12-08

**Authors:** Tamara Veiga-Parga, Amol Suryawanshi, Barry T. Rouse

**Affiliations:** 1 Department of Pathobiology, College of Veterinary Medicine, University of Tennessee, Knoxville, Tennessee, United States of America; 2 Emory Vaccine Center and Yerkes Primate Research Center, Atlanta, Georgia, United States of America; University of Pennsylvania, United States of America

## Abstract

Ocular herpes simplex virus infection can cause a blinding CD4^+^ T cell orchestrated immuno-inflammatory lesion in the cornea called Stromal Keratitis (SK). A key to controlling the severity of SK lesions is to suppress the activity of T cells that orchestrate lesions and enhance the representation of regulatory cells that inhibit effector cell function. In this report we show that a single administration of TCDD (2, 3, 7, 8- Tetrachlorodibenzo-p-dioxin), a non-physiological ligand for the AhR receptor, was an effective means of reducing the severity of SK lesions. It acted by causing apoptosis of Foxp3^-^ CD4^+^ T cells but had no effect on Foxp3^+^ CD4^+^ Tregs. TCDD also decreased the proliferation of Foxp3^-^ CD4^+^ T cells. The consequence was an increase in the ratio of Tregs to T effectors which likely accounted for the reduced inflammatory responses. In addition, *in vitro* studies revealed that TCDD addition to anti-CD3/CD28 stimulated naïve CD4^+^ T cells caused a significant induction of Tregs, but inhibited the differentiation of Th1 and Th17 cells. Since a single TCDD administration given after the disease process had been initiated generated long lasting anti-inflammatory effects, the approach holds promise as a therapeutic means of controlling virus induced inflammatory lesions.

## Author Summary

This report describes a novel approach to control a blinding immuno-inflammatory reaction in the eye caused by herpes simplex virus. We showed that a single administration of TCDD, a stable agonist of the aryl hydrocarbon receptor, significantly reduced the severity of herpes keratitis lesions. The outcome of the therapy was a change in the balance of effector cells responsible for orchestrating lesions, with regulatory cells able to inhibit the inflammatory effects of the effectors. Since a single administration of TCDD provided effective treatment that lasted for as long as one month, this approach could represent a valuable therapy for a lesion that is a common cause of human blindness.

## Introduction

Ocular infection with herpes simplex virus (HSV) can result in a chronic immuno-inflammatory reaction in the cornea which represents a common cause of human blindness [1], [2]. The pathogenesis of stromal keratitis (SK) involves numerous events, but studies in murine SK models indicate that lesions are mainly orchestrated by CD4^+^ T cells that recognize virus derived peptides, or perhaps altered self proteins unmasked in the damaged cornea [1]–[4]. The severity of SK can be influenced by the balance of CD4^+^ effector T cells and Foxp3^+^ regulatory T cells (Treg) [5], [6]. Procedures that change this balance represent a promising approach for therapy. This has been achieved either by adoptive transfer with Treg populations [6] or the repeated administration of reagents that can cause naïve CD4^+^ T cells to convert to become Treg [7], [8]. From a therapeutic angle, procedures that could shift the balance of T effectors and Treg after a single drug administration would represent a convenient maneuver.

Recent evidence from studies to control autoimmunity and graft-versus-host disease indicate that the objective might be achieved by the administration of stable agonists of the aryl hydrocarbon receptor (AhR) [9]–[11]. The AhR is a cytosolic transcription factor that can be activated by different ligands. These include the physiological ligand tryptophan photoproduct 6-formylindolo(2,3-b)carbazole (FICZ), and synthetic molecules such as 2, 3, 7, 8- tetrachlorodibenzo-p-dioxin (TCDD) [10], [12]. Signaling through the AhR has consequences that include changes in innate cell function, as well as some modulatory effects on several aspects of T cell immunity [13], [14]. For example, Weiner and colleagues showed that TCDD administration could suppress the induction of experimental autoimmune encephalomyelitis (EAE), an effect attributed to a reduction of proinflammatory T cells along with the expansion of Treg [9]. By a similar mechanism, TCDD had suppressive effects in an autoimmune diabetes model [15]. Similarly, the administration of TCDD prior to the induction of colitis led to reduced lesions along with an increase in the Treg population [16]. In graft versus host disease (GVHD) too, the reduced lesions in TCDD treated animals was attributed to the expansion of adaptive Tregs that suppressed allospecific cytotoxic T cell generation [11], [17]. Modulating AhR by TCDD has also been shown to control the differentiation of Type 1 regulatory T cells (Tr1) *in vitro*, which produce IL-10 and are instrumental in the prevention of tissue inflammation, autoimmunity as well as GVHD [18].

Although AhR ligation can result in reduced inflammatory lesions, in some circumstances lesions may be exacerbated. This was noted in the Weiner studies when the physiological ligand FICZ, rather than TCDD, was used for treatment [9]. In this study administration of FICZ boosted Th17 differentiation and increased the severity of EAE. Proinflammatory effects of AhR activation were also noted in a model of rheumatoid arthritis [19], where synoviocytes were exposed to different concentrations of TCDD and shown to produce inflammatory cytokines. Additional proinflammatory effects of AhR ligation were also associated with pulmonary neutrophilia [20], [21] **,** as well as with the induction and expansion of IL-17^+^ secreting CD4^+^ T cells (Th17) that expressed high levels of AhR receptors [22], [23]. Currently, it is not clear why AhR activation causes either an increased, or a reduced effect on inflammatory reactions, but the stability of the ligand used for AhR stimulation is one suspected explanation [24]. Accordingly, TCDD is a non-degradable high affinity ligand for AhR and most studies using this ligand report inhibitory effects on inflammatory reactions [24], [25].

The effects of AhR agonists have not been evaluated in microbe induced inflammatory lesions. In this report, we show that a single administration of the stable AhR ligand TCDD was highly effective at suppressing the severity of ocular immuno-inflammatory lesions caused by HSV. The outcome was attributed to inhibitory effects on inflammatory IFN-γ^+^ secreting CD4^+^ T cells (Th1) and Th17 cells. However, since Foxp3^+^ regulatory T cell numbers remained unchanged by the treatment, the balance between T effectors and Tregs favored the latter population. TCDD was also shown to cause apoptosis *ex vivo* of Foxp3^-^ CD4^+^ T cells and could cause some naïve T cells to convert to Foxp3^+^ CD4^+^ T cells. Since a single TCDD administration given after the disease process had been initiated generated long lasting anti-inflammatory effects, the approach holds promise as a therapeutic means of controlling virus induced inflammatory lesions.

## Results

### Modulation of AhR signaling diminishes HSV-1 mediated immunopathology

**Figure 1:**
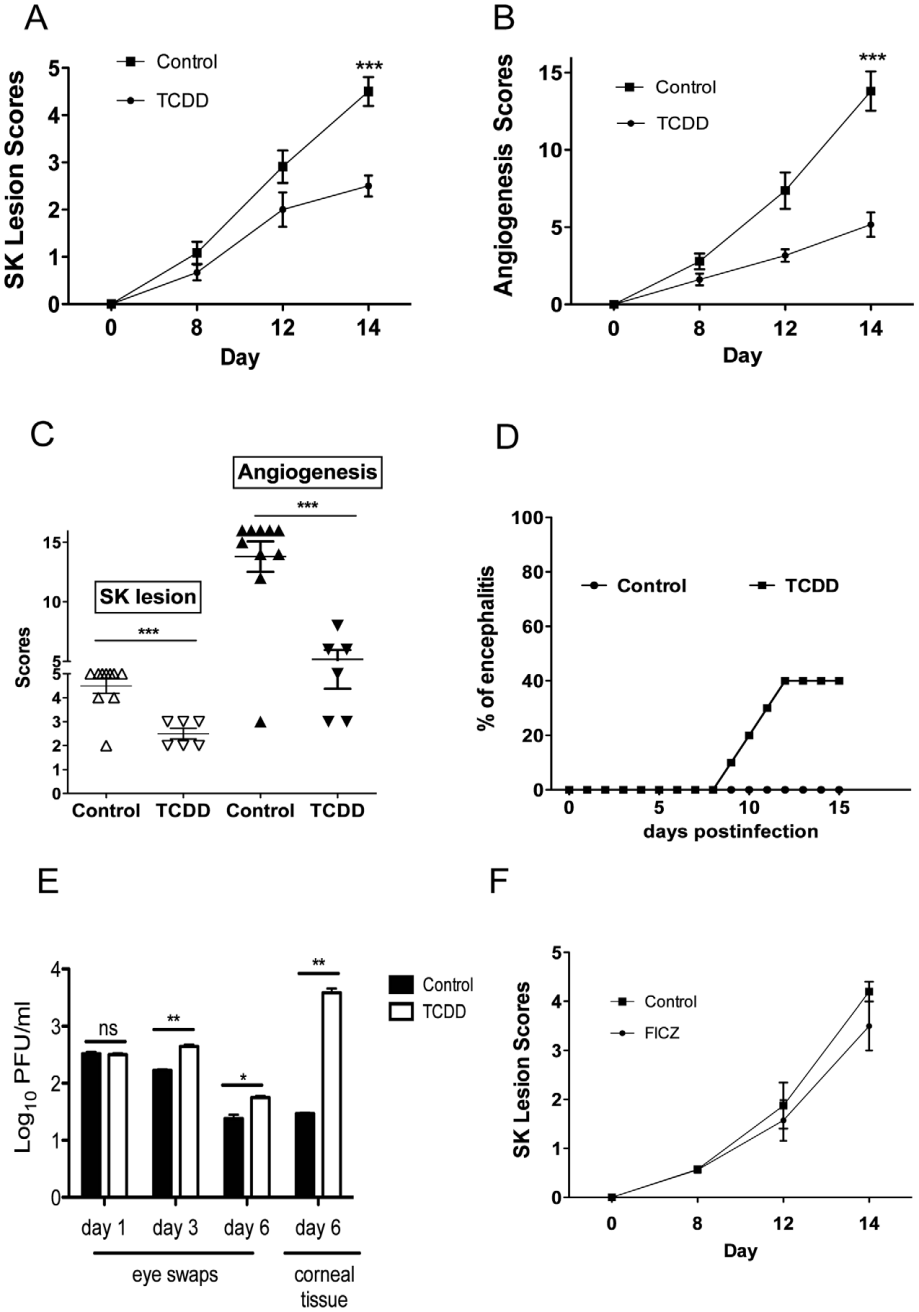
Preventive administration of TCDD diminishes SK severity. C57BL/6 animals infected with 1 x 10^4^ PFU of HSV were given either TCDD or vehicle IP on day 1 pi or FICZ or vehicle IP from day 1 to day 11 pi. The disease progression was analyzed throughout time in a blinded manner using a scale described in materials and methods. A, The progression of SK lesion severity was significantly reduced in the group of mice treated with TCDD as compared with control mice. Kinetics of SK severity is shown. B, The progression of angiogenesis was significantly reduced in the group of mice treated with TCDD as compared with control mice. Kinetics of angiogenesis lesion severity is shown. C, Individual eye scores of SK lesion severity and angiogenesis on day 14 pi. D, Percentage of encephalitis through time in control and TCDD treated mice. Herpetic encephalitis is an acute inflammation of the brain caused by HSV-1 and in mice gives clinical signs such as lethargy, ruffled fur, and hind limb paralysis [54], [55]. E, Eye swabs were taken from infected corneas from treated and control animals using sterile swabs on day 1, 3 and 6 pi or corneal tissue on day 6 pi and titration was performed by standard plaque assay as described on materials and methods. Titers were calculated as log_10_ pfu/ml. F, Kinetics of SK lesion scores in mice treated with FICZ or vehicle. Data are representative of 3 independent experiments and show mean values ± SEM (n = 10 mice/group). *P*≤0.001(***), *P*≤0.01(**), *P*≤0.05(*).

To evaluate the role of AhR engagement on the outcome of ocular HSV infection, mice were given a single intraperitoneal (IP) administration of TCDD on day 1 post-infection (pi), and the effect on the severity of ocular lesions was compared to untreated controls. All treated animals developed significantly reduced lesions compared to controls, but around 40% of the animals developed clinical signs typical of herpes encephalitis before the end of the 15 day observation period and had to be terminated (Figure 1A–D). Ocular viral loads were also increased in the TCDD treated group (Figure 1E). Accordingly, the drug was judged to be effective but would not be recommended for use when virus is present and actively replicating in the cornea. In other experiments, the physiological AhR ligand FICZ was administered daily starting at day 1 pi. This drug was without significant effects on lesion severity (Figure 1F), and none of the treated animals developed herpetic encephalitis (data not shown).

**Figure 2:**
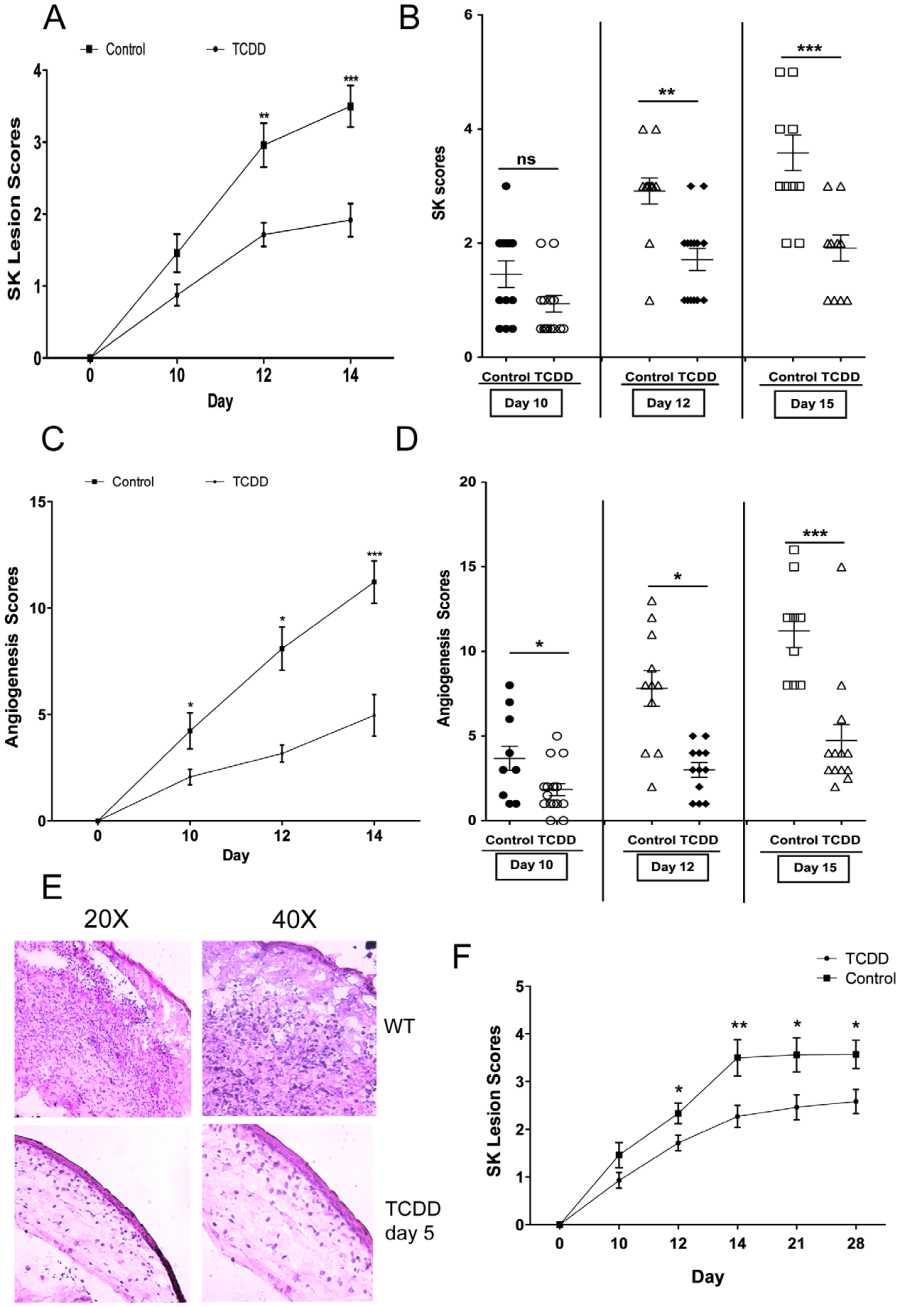
Therapeutic administration of TCDD diminishes SK severity. C57BL/6 animals infected with 1×10^4^ PFU of HSV were given either TCDD or vehicle IP on day 5 pi. The disease progression was analyzed throughout time. A, The progression of SK lesion severity was significantly reduced in the group of mice treated with TCDD as compared with control mice. Kinetics of SK severity is shown. B, Individual eye SK scores on day 10, 12 and 15 pi. C, The progression of angiogenesis was significantly reduced in the group of mice treated with TCDD as compared with control mice. Kinetics of angiogenesis lesion severity is shown. D, Individual eye angiogenesis scores on day 10, 12 and 15 pi. E, Eyes were processed for cryo-sections on day 15 pi. Hematoxylin and eosin staining was carried out on 6-μm sections and pictures were taken at different microscope augmentations. F, The progression of SK lesion severity was significantly reduced in the group of mice treated with TCDD as compared with control mice. Kinetics of SK severity is shown up to day 28 pi. Data are representative of 3 independent experiments and show mean values ± SEM (n = 14 mice/group). *P*≤0.001(***), *P*≤0.01(**), *P*≤0.05(*).

In additional experiments, TCDD administration was begun on day 5 pi, a time when levels of replicating virus in the cornea were barely detectable and inflammatory lesions start to become evident [3]. This treatment procedure resulted in significantly reduced lesion severity, as well as the extent of corneal neovascularization, compared to untreated infected controls (Figure 2A–D), and none of the treated animals developed encephalitis. The treatment procedure delayed the time of lesion appearance and average severity scores were significantly less at most time points over a 15 day observation period. For example, on day 12 pi, whereas 10 of 12 eyes from untreated animals had lesion scores of 3 or above, only 2 of 14 eyes in the treated group had lesions of such severity (Figure 2B). An example of comparative severity of control and treated animals is shown in the histological sections in Figure 2E. In additional experiments terminated on day 28 pi, the pattern of results was similar with treated animals showing significantly diminished lesions compared to untreated controls (Figure 2F). In conclusion, ligation of the AhR with a single administration of TCDD given 5 days after virus infection significantly diminished HSV induced immunopathology.

### Modulation of AhR signaling diminishes cell infiltration as well as cytokines after HSV-1 infection

**Figure 3:**
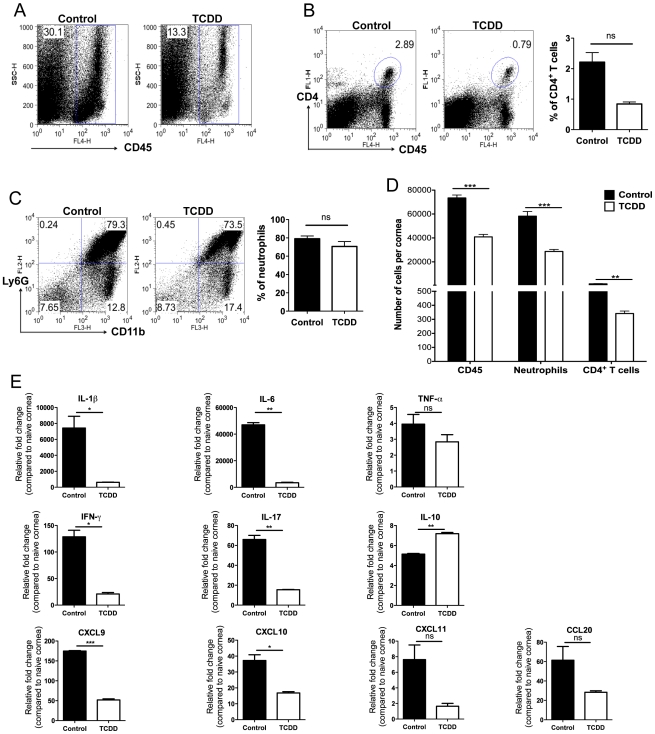
TCDD treatment reduces cellular infiltration and cytokine levels in corneas of HSV infected animals. Wild-type C57BL/6 mice corneas were scarified and infected with 1×10^4^ PFU of HSV-1 in PBS and mice were given either TCDD or vehicle IP on day 5 pi. A, Representative FACS plots of CD45^+^ cells infiltrated in the corneas of control (left) and day 5 TCDD treated (right panel) mice are shown. B, Representative FACS plots and percentages of CD4^+^ T cells gated on total CD45^+^ cells infiltrated in the corneas of control (left) and day 5 TCDD treated (right panel) mice are shown. C, CD11b^+^ Ly6G^+^ polymorphonuclear neutrophils gated on total CD45^+^ cells infiltrated in the corneas of control (left panel) and day 5 TCDD treated (right panel) mice are shown. D, Numbers of total CD45^+^ cells, neutrophils gated on total CD45^+^ cells and CD4^+^ T cells gated on total CD45^+^ cells infiltrated per cornea of control and TCDD treated mice. Data are the combination of 3 independent experiments and show mean values ± SEM (n = 9 and each sample is representative of 2 corneas). Total number of CD45^+^ cells recovered from corneal samples varied and ranged from around 20,000 to 80,000 cells. E, Relative fold change in the mRNA expression of various cytokines (IL-1β, TNF-α, IL-6, IFN-γ, IL-17, IL-10, CXCL9, CXCL10, CXCL11, CCL20) was examined and compared between control and TCDD day 5 treated mice on day 15 pi by Q-RTPCR. mRNA levels for the different cytokines in mock infected mice were set to one and used for the relative fold upregulation. Data represents means ± SEM from two different independent experiments (n = 2 and each sample is representative of 8 corneas). Day 15 pi corneal samples were used for all the experiments. *P*≤0.001(***), *P*≤0.01(**), *P*≤0.05(*).

To measure the effect of TCDD treatment on the cellular composition of SK lesions collagen digested corneas were analyzed by FACS and compared to controls at day 15 pi. The combination of three independent experiments is shown in Figure 3A–D. As shown in Figure 3D, the average number per cornea of neutrophils and CD4^+^ T cells was reduced in the treated group by 2.03 fold and 4.7 fold respectively when compared to untreated controls.

In separate experiments of the same design, pools of corneas were processed to quantify mRNA of selected cytokines (IL-1β, TNF-α, IL-6, IFN-γ, and IL-17) and chemokines (CCL20, CXCL9, CXCL10, and CXCL11) by quantitative real time PCR (Q-RTPCR). As shown in Figure 3C, the consequence of TCDD treatment was a reduction in the levels of several proinflammatory cytokines and chemokines. However, levels of the cytokine IL-10 was increased to 1.4 fold in samples from treated compared to controls. Taken together, our results show AhR ligation by TCDD significantly reduced the total cellular infiltration of CD4^+^ T cells and neutrophils, as well as the amount of proinflammatory cytokines and chemokines.

### AhR signaling changes the balance of effectors and Treg

**Figure 4:**
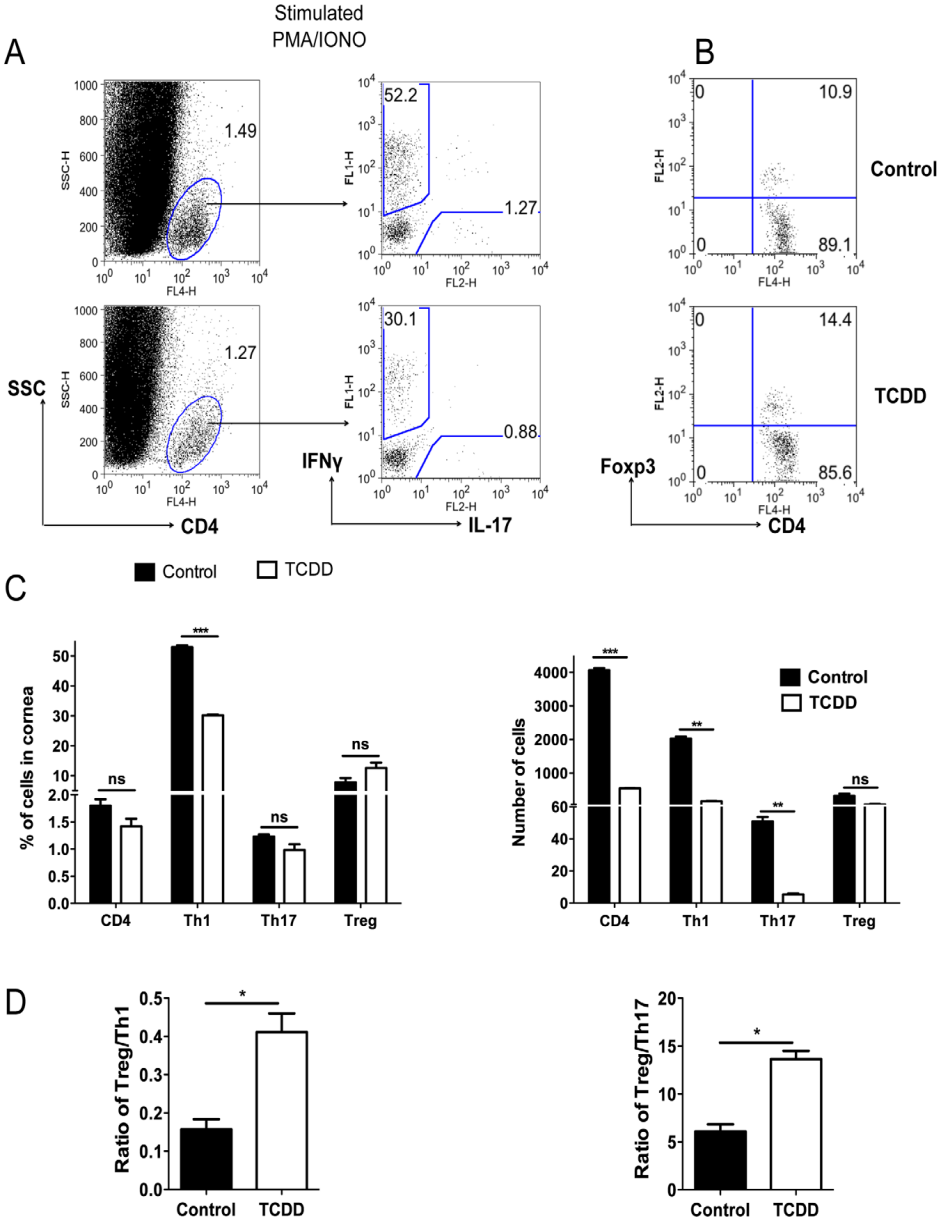
TCDD treatment shifts the balance from T effectors to T regulatory cells after HSV infection. C57BL/6 animals infected with 1×10^4^ PFU of HSV were given either TCDD or vehicle IP on day 5 pi. Mice were sacrificed and corneas were collected on day 15 pi. A, Representative FACS plots gated on CD4^+^ T cells for IFN-γ and IL-17 secreting cells from pooled corneas stimulated with PMA/ionomycin during 4 hours from control and day 5 TCDD treated mice. B, FACS plots of Foxp3^+^CD4^+^ cells from pooled corneas from control and day 5 TCDD treated mice. C, Frequencies and total numbers of CD4^+^ T cells, Th1, Th17 and Tregs in infected corneas from control and day 5 TCDD treated animals. D, Cell ratios for total numbers of Tregs per Th1 cell and Tregs per Th17 cell. Data are representative of 3 independent experiments and show mean values ± SEM (n = 6 and each sample is representative of 2 corneas). *P*≤0.001(***), *P*≤0.01(**), *P*≤0.05(*). Day 15 pi corneal samples were used for the experiments.

To measure the consequences of TCDD treatment on the T cell subset composition of SK lesions at day 15 pi, pools of corneas from treated and control animals were collagen digested to recover the T cell population. Part of the pool was stimulated in vitro for 4 hours with PMA and ionomycin to enumerate cells that were either IFN-γ or IL-17 producers. The other fraction was used to enumerate Foxp3^+^ CD4^+^ T cells. In the experiment shown, there was an average 12.3 fold reduction of Th1 cells and a 9.4 fold reduction of Th17 cells in treated compared to control corneas. The numbers of Foxp3^+^ T cells were almost identical in corneal pools from treated and control animals (Figure 4C). Two additional experiments provided a similar pattern of results. Taken together, our results show that a consequence of TCDD treatment was to increase the ratio of total numbers of Foxp3^+^ CD4^+^T cells to both, Th1 and Th17 cells (Figure 4D).

**Figure 5:**
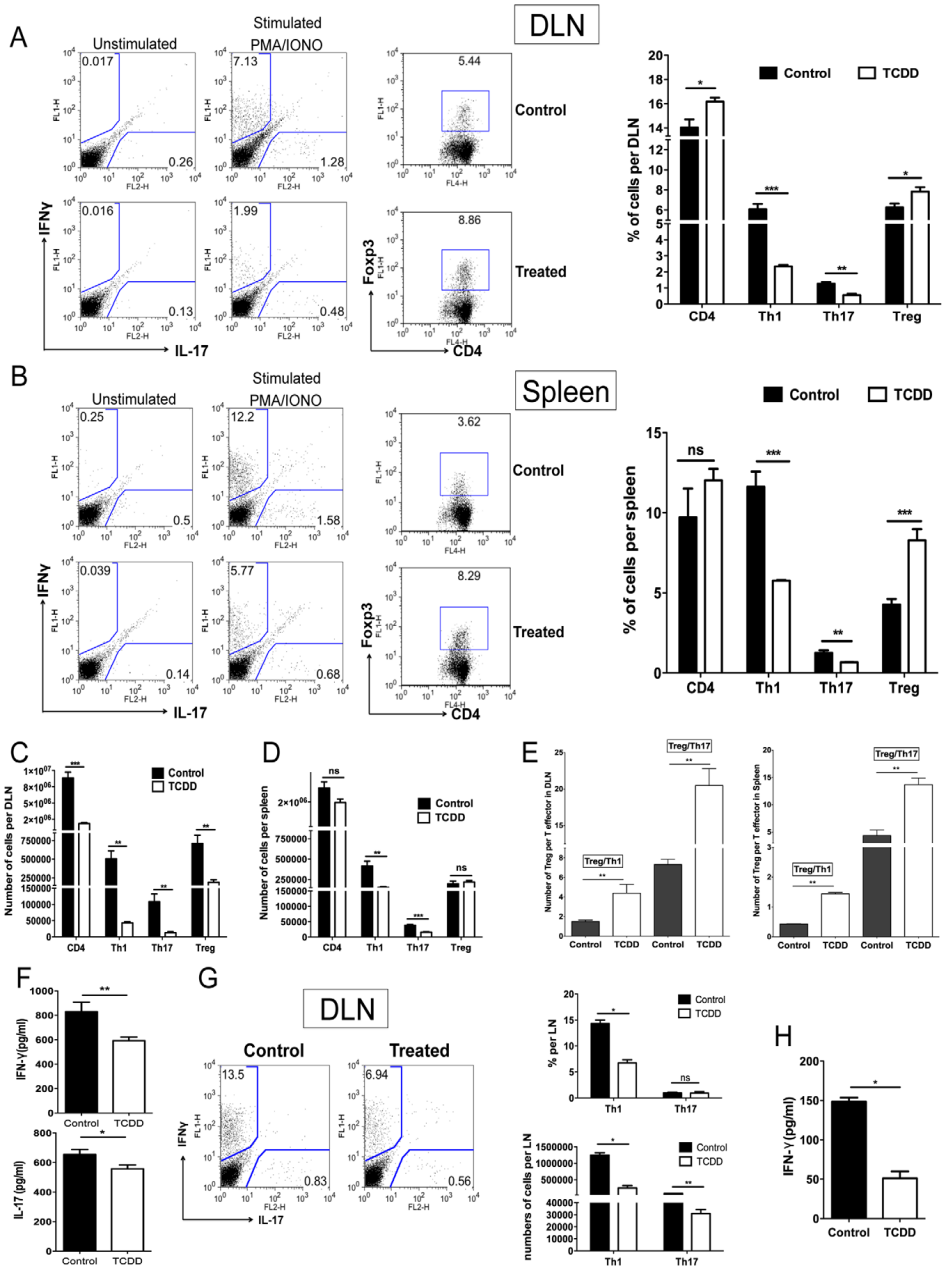
Differential effect of TCDD treatment on Treg and effector T cells in lymphoid organs of TCDD treated animals. C57BL/6 animals infected with 1×10^4^ PFU of HSV were given either TCDD or vehicle IP on day 5 pi. A, Representative FACS plots and percentages for IFN-γ and IL-17 secreting T cells gated on CD4^+^ T cells from day 15 DLN stimulated with PMA/ionomycin during 4 hours from control and day 5 TCDD treated mice. FACS plots of CD4^+^Foxp3^+^ cells from DLN from control and day 5 TCDD treated mice. B, Representative FACS plots and percentages for IFN-γ and IL-17 secreting T cells gated on CD4^+^ T cells from day 15 spleens stimulated with PMA/ionomycin during 4 hours from control and day 5 TCDD treated mice. FACS plots of CD4^+^Foxp3^+^ cells from spleens from control and day 5 TCDD treated mice. C, Cell numbers of CD4, Th1, Th17 and Tregs in DLNs stimulated with PMA/ionomycin. D, Cell numbers of CD4, Th1, Th17 and Tregs in spleen stimulated with PMA/ionomycin. E, Cell ratios for total number of Tregs per Th1 cell and Tregs per Th17 cell in DLN and spleen. F, IFN-γ and IL-17 protein levels analyzed by ELISA from control and TCDD treated infected DLN on day 15 pi. G, FACS plots, percentages and cell numbers for IFN-γ and IL-17 secreting T cells gated on CD4^+^ T cells from day 10 DLN samples, purified for CD4^+^ T cells and stimulated with PMA/ionomycin during 4 hours. H, IFN-γ protein levels analyzed by ELISA from control and TCDD treated infected DLN at day 10 pi, purified for CD4^+^ T cells and stimulated with PMA/ionomycin during 4 hours. Data are representative of 3 independent experiments and show mean values ± SEM (n = 12). *P*≤0.001(***), *P*≤0.01(**), *P*≤0.05(*).

Parallel studies of a similar design were performed with T cells isolated from the draining lymph nodes (DLN) and spleen collected from the same animals used for the corneal studies. The results shown in Figure 5A–D demonstrate that Th1 and Th17 cell frequencies and total numbers per organ were significantly reduced in TCDD recipients when compared to controls. However the frequencies of Foxp3^+^ Tregs, compared as a fraction of total CD4^+^ T cells, were increased in treated animals when compared to controls. Additionally, when the ratio of total numbers of Treg per T effectors was compared to controls, a significant increase in the number of Treg per Th1 or Th17 cells in the TCDD treated mice was evident (Figure 5E).

To compare levels of IFN-γ and IL-17 produced by CD4^+^ T cells from infected and treated or untreated mice, sorted CD4^+^ T cells were isolated from DLN on day 10 pi and stimulated in vitro with PMA and ionomycin. When comparing the number of IFN-γ and IL-17 secreting CD4^+^ T cells by ICCS, averages were reduced for both in TCDD treated animals (Figure 5G). Similarly IFN-γ secreting levels measured by ELISA were reduced 2.9 fold as a consequence of TCDD treatment (Figure 5H).

### AhR signaling promotes Treg induction but suppresses Th1 and Th17 cell generation in vitro

**Figure 6:**
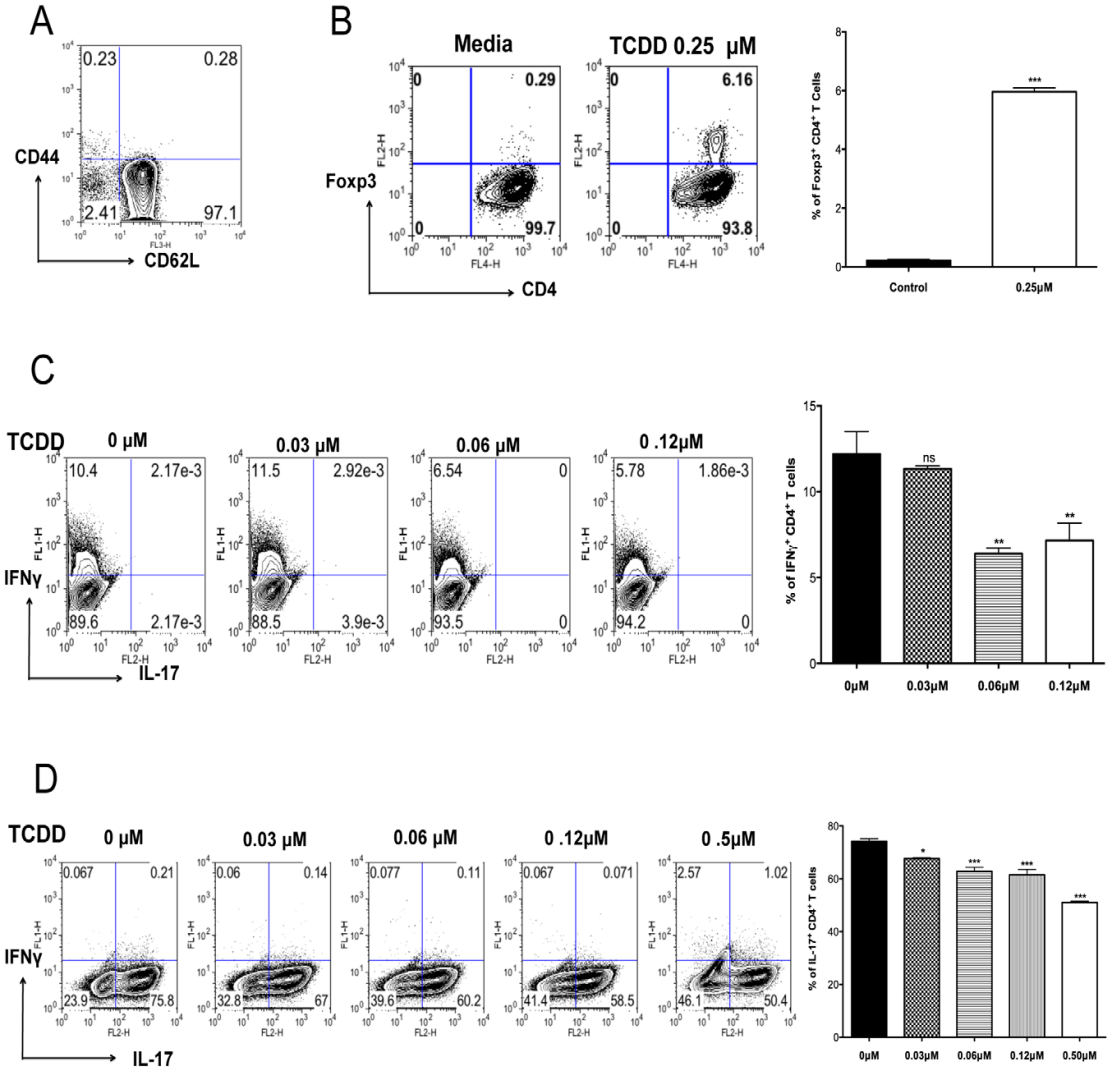
TCDD promotes Treg differentiation whereas diminishes Th1 and Th17. Naïve splenocytes obtained from DO11.10RAG2^-/-^ mice were differentiated under different conditions for 5 days for the *in vitro* generation of different T cell subsets followed by ICS of Foxp3^+^, IFN-γ, and Il-17. A, Representative FACS plots of splenocytes stained for CD62L and CD44 gated on CD4^+^ T cells. B, Representative FACS plots showing the induction of Foxp3^+^CD4^+^ T cells under the indicated incubation conditions. Bar diagram showing the percentages of Foxp3^+^CD4^+^ T cells under the indicated conditions C, Frequencies of IFN-γ^+^CD4^+^ T cells differentiated in the presence of TCDD at different concentrations. Bar diagram showing the percentages of IFN-γ^+^CD4^+^ T cells under the indicated conditions. D, Frequencies of IL-17^+^CD4^+^ T cells differentiated in the presence of TCDD at different concentrations. Bar diagram showing the percentages of IL-17^+^CD4^+^ T cells under the indicated conditions Experiments were repeated at least 3 times. Data were analyzed using one-way ANOVA test with Dunett's post hoc test settings and show mean values ± SEM. *P*≤0.001(***), *P*≤0.01(**), *P*≤0.05(*).

Results from the previous section indicated that there was a shift in the balance between Tregs and T effectors towards Tregs, as well as a reduction in the production of proinflammatory cytokines. To further determine how TCDD could change the balance of Treg to T effectors, naïve splenocytes from DO11.10RAG2^-/-^ (98% naïve CD4^+^ T cells) animals were stimulated *in vitro* with plate bound anti-CD3 and anti-CD28, in the presence of IL-2. Cultures were either untreated or treated with graded amounts of TCDD (from 0.1 µM to 0.25 µM). Cultures with 0.25 µM of TCDD significantly triggered the conversion of approximately 6.2% of CD4^+^ T cells into Foxp3^+^ CD4^+^ T cells, as compared to 0.3% in the untreated controls (Figure 6B).

Other cultures were TCR stimulated in a cytokine cocktail reported to induce either Th1 or Th17 cells in the additional presence of different doses of TCDD. The outcome was a significant decrease in both Th1 and Th17 cell induction (Figure 6C–D) with the highest TCDD dose studied (0.6 µM) causing a disappearance of the majority CD4^+^ T cells from the cultures (data not shown). Taken together, our results indicate that activation of AhR signaling by TCDD can induce some CD4^+^ T cells to become Foxp3^+^, but it is inhibitory to the generation of IFN-γ^+^ CD4^+^ and IL-17^+^ CD4^+^ T cells.

### Differential proliferation of Treg over Foxp3^-^ CD4^+^ T cells in TCDD treated mice

**Figure 7:**
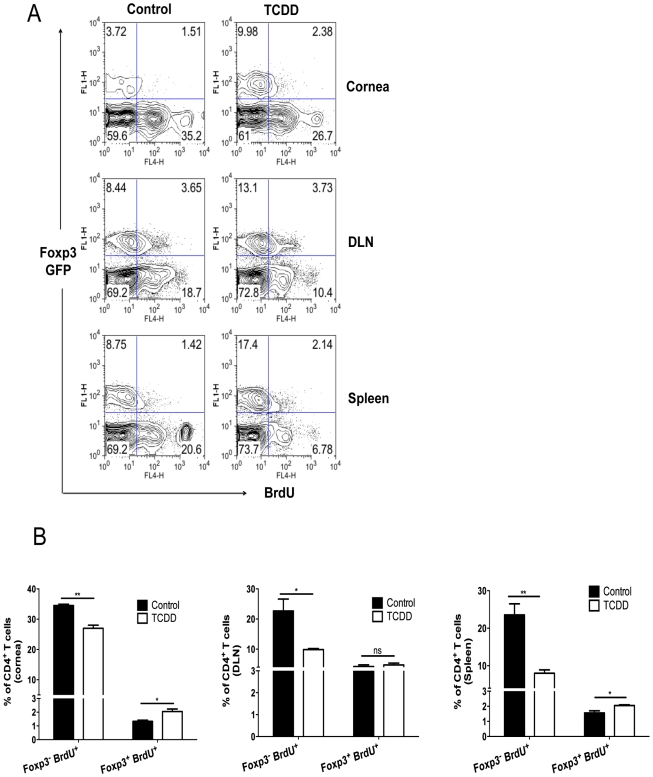
Differential proliferation of Treg over Foxp3^-^CD4^+^ T cells in TCDD treated mice after HSV-1 infection. A, Control and day 5 TCDD treated GFP-Foxp3^+^ mice were given BrdU intraperitoneally one day before termination and the next day (day 9 pi) corneas, DLN and spleens were analyzed for the frequencies of Foxp3^+^CD4^+^ and Foxp3^-^ CD4^+^ T cells that incorporated BrdU; representative FACS plots for cells are shown. B, The bar diagram shows the frequencies of Treg and Foxp3^-^CD4^+^ T cells labeled for BrdU in cornea, DLN and spleen. Data are representative of 3 independent experiments and show mean values ± SEM (n = 12). *P*≤0.001(***), *P*≤0.01(**), *P*≤0.05(*).

To determine if TCDD had differential effects on Foxp3^+^ and Foxp3^-^ CD4^+^ T cell proliferation, Foxp3-GFP mice were infected and some treated with TCDD on day 5 pi. After an injection of 5-Bromo-2-deoxyuridine (BrdU) on day 8 pi, experiments were terminated on day 9 pi and proliferation of both Foxp3^-^CD4^+^ and Foxp3^+^ cells was detected by BrdU incorporation. Our results show that TCDD treatment significantly reduced the proliferative response of the Foxp3^-^ CD4^+^ T cell population in both corneas and lymphoid tissue, but was without significant inhibitory effects on the Foxp3^+^ CD4^+^ T cell population. Instead, the effect of TCDD on Foxp3^+^ cells in the cornea was to cause a modest increase in proliferation (Figure 7A–B). These effects could explain in part the balance between Tregs and T effectors in corneal lesions.

### Induction of apoptosis of Foxp3^-^ CD4^+^ T cells but not of Treg after TCDD treatment

**Figure 8:**
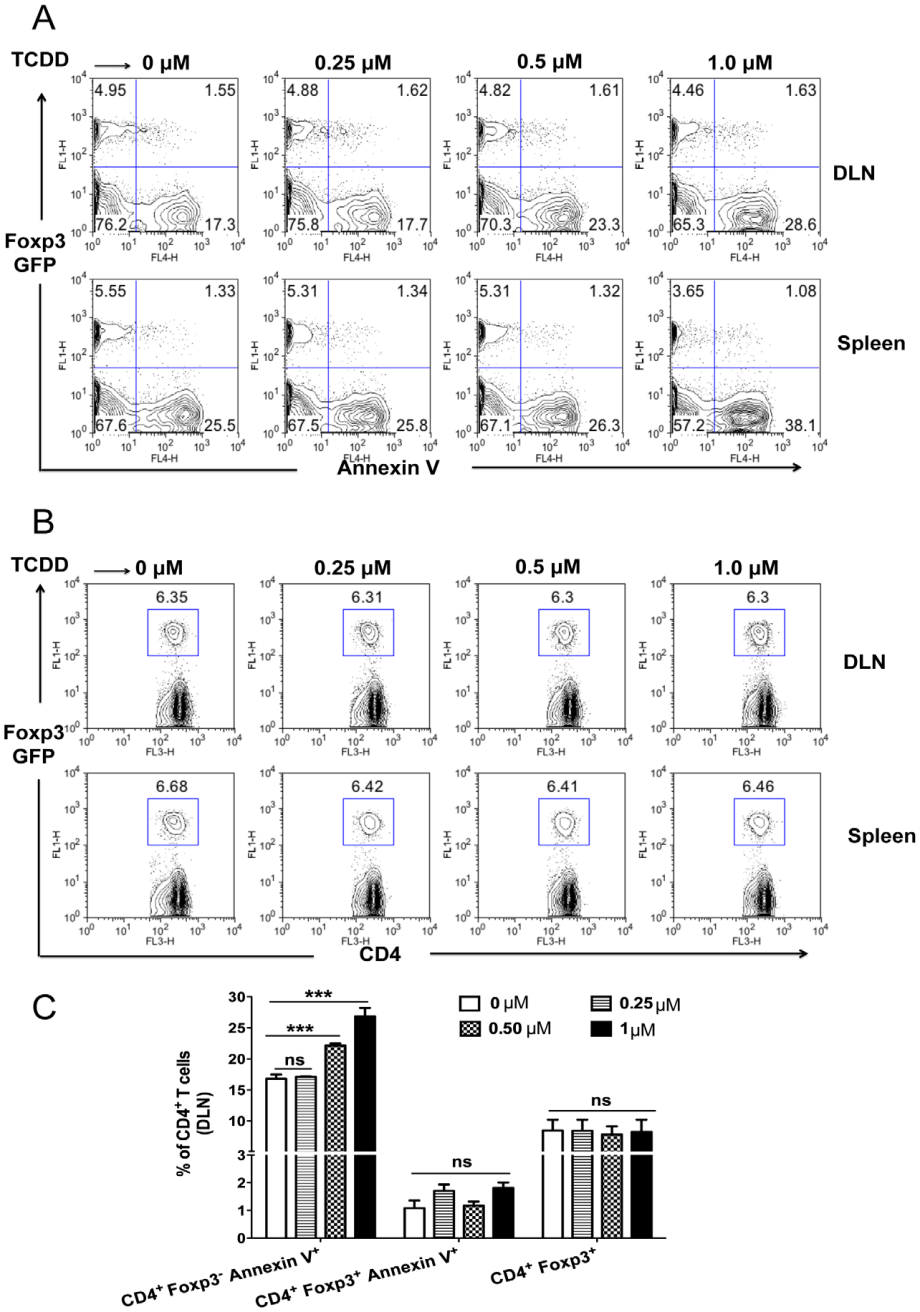
TCDD causes apoptosis of Foxp3^-^CD4^+^ T cells but not Tregs. Foxp3-GFP mice were HSV-1 infected and sacrificed on day 8 pi. A, Induction of *in vitro* apoptosis by different concentrations of TCDD on Foxp3^-^CD4^+^ T cells but not on Foxp3^+^CD4^+^ T cells. Cells isolated from spleens and DLNs of HSV-1 infected mice were cultured *ex vivo* in the presence of RPMI or RPMI with different concentrations of TCDD for 5 hours and thereafter stained for Annexin-V, representative FACS plots showing Annexin-V expression on gated CD4^+^ T cells. B, Representative FACS plots of Foxp3^+^CD4^+^ T cells in splenocytes and DLN cells from HSV-1 infected mice treated *in vitro* with different concentrations of TCDD for 5 hours. C, Bar diagram showing the percentages of Annexin V^+^Foxp3^-^CD4^+^, Annexin V^+^Foxp3^+^CD4^+^ and Foxp3^+^CD4^+^ under the indicated conditions in the DLN is shown. Data are representative of 3 independent experiments. Data were analyzed using one-way ANOVA test with Dunett's post hoc test settings and show mean values ± SEM (n = 12). *P*≤0.001(***), *P*≤0.01(**), *P*≤0.05(*).

Prior studies had shown that TCDD administration *in vivo* causes thymocytes to undergo apoptosis [26]. We determined if apoptosis of Foxp3^-^ CD4^+^ T cells could account for the reduced numbers of T effectors. We performed experiments with CD4^+^ T cells isolated from DLN or spleen on day 8 pi from HSV infected Foxp3-GFP mice. Cells were cultured *ex vivo* in the presence of TCDD for 5 hours and apoptosis of Foxp3^-^ and Foxp3^+^ CD4^+^ T cells was measured using Annexin-V staining. The result showed a dose dependent increase in the apoptosis of Foxp3^-^CD4^+^ T cells, but no significant apoptosis of Foxp3^+^CD4^+^ T cells (Figure 8A and C). Notably, there was no difference in the frequencies of Tregs with the addition of different concentrations of TCDD as compared to media (Figure 8B–C). Taken together, these results indicate that AhR signaling by TCDD, can promote the apoptosis of Foxp3^-^ CD4^+^ T cells in vitro, but did not cause the same effect in Foxp3^+^ Treg.

## Discussion

SK is a blinding immuno-pathological lesion induced by ocular infection with HSV [1]-[3]. Novel treatment procedures are needed to replace the current long term use of antivirals and corticosteroids which have unwanted side effects [27]–[29]. A key to controlling the severity of SK lesions is to suppress the activity of T cells that orchestrate lesions and enhance the representation of cellular and humoral events that inhibit effector cell function. In this report, we have evaluated the use of a novel approach to achieve lesion control in a murine model of SK. We demonstrate that modulation of AhR signaling with a single dose of a synthetic stable molecule (TCDD) causes cellular changes in the cornea after HSV infection that account for significantly reduced SK lesion severity. The outcome of therapy was reduced effector Th1 and Th17 cells that orchestrate lesions, a reduction of neutrophils that are mainly responsible for damage to the cornea, as well as an increase in the representation of Foxp3^+^ Treg. Accordingly, when the ratio of Treg per T effectors was compared to controls, a significant increase in the number of Treg per Th1 as well as Th17 cells in the TCDD treated mice was evident. Foxp3^+^CD4^+^ T cells are assumed to function by inhibiting the inflammatory effects of T effectors either directly, or by the generation of counter inflammatory molecules [30]. Since a single administration of TCDD provided effective treatment that lasted for as long as one month, this approach could represent an effective novel therapy for a lesion that is a common cause of human blindness.

Aryl hydrocarbon receptors are found in animals in many levels of the evolutionary scale. They can recognize numerous low molecular weight synthetic chemicals as well as a list of endogenous ligands, some of which are photoproducts of tryptophan breakdown [24], [31]. Several cell types express AhR that includes some, but not all cells, involved in innate and adaptive immunity [32]. Our own interest in AhR ligands stemmed from recent reports that synthetic AhR agonists had anti-inflammatory activity [9], [15]. Moreover, the dioxin TCDD can provide long term activation of the AhR since it is resistant to metabolic degradation [25]. As a consequence, a single administration can result in long term effects on immune mediated diseases. A recent report using the animal model of multiple sclerosis, EAE, showed disease suppression when animals were pretreated with TCDD [9]. The diminished lesions in treated animals were correlated with expansion of the Foxp3^+^ CD4^+^ T cell population and the levels of some cytokines produced by effector cells were reduced. The expansion of the Treg population was explained in part by conversion of naïve T cells to become Treg as could be demonstrated *in vitro*. In our studies too, we observed that a single TCDD administration was an effective means of reducing HSK ocular lesions, but with our model the outcome appeared to be more the consequence of suppressed numbers of Th1 and Th17 T cells that orchestrate SK, than any notable effect on the expansion of Tregs. Accordingly, the cytokine producing cells in lesions were reduced several fold in treated animals, whereas Treg numbers remained approximately the same in treated and controls. We did confirm the Weiner group [9] observations that TCDD can cause some naïve T cells to convert and become Foxp3^+^ *in vitro*, but in our hands this was a modest effect. This notwithstanding, it could be that the relative increase in Treg in the SK lesions of treated animals was the explanation for the reduced lesions, the Treg acting by inhibiting the functions of effectors as well as producing anti-inflammatory cytokines such as IL-10.

Equally possible, however, was that the reduced lesions were the direct consequence of the fewer numbers and less functional effectors in the corneas of treated animals. Such effectors would be less able to recruit inflammatory cells such as neutrophils that are considered responsible for much of the tissue damage of SK [33], [34]. The reduced numbers of effectors would likely arise either, or both, from an inhibitory effect of TCDD on effector cell proliferation and differentiation, or be explained by the drug causing apoptosis of effectors. The latter effect could readily be demonstrated by *in vitro* studies with TCR stimulated CD4^+^ T cells cultured with TCDD. In addition, Foxp3^-^ CD4^+^ T cells from treated animals proliferated less *in vivo* than did cells from control animals. In some reports TCDD was shown to prematurely terminate the proliferation and decrease the survival of CD4^+^ T cell, although differential effects on T cell subsets were not investigated [35]. Nevertheless, since regulatory T cells may be more resistant to apoptosis than conventional T cells [32], [36] frequencies of Tregs would be expected to increase when other T cell populations are depleted.

The reduced number of effector cells present in drug treated animals in our studies were also functionally impaired in their ability to mediate inflammatory reactions. Accordingly, *ex vivo* stimulation of DLN cells from drug-treated mice produced lower levels of some proinflammatory cytokines as well as chemokines responsible for neutrophil recruitment than cells from control animals. During in *vitro studies*, AhR ligation was shown to affect the differentiation of T helper subsets, behaving differently under identical culture conditions depending on the ligand used. TCDD for example, was shown to trigger the conversion of Foxp3^-^CD4^+^ into Foxp3^+^CD4^+^ without the need for TGF-β addition [9], [37], to dampen Th17 differentiation [37] and to increase the frequency of IL-17 secreting cells induced by TGF-β plus IL-6 [38]. On the other hand, FICZ under identical culture conditions promoted Th17 differentiation, but not Treg differentiation [37], [38]. Other ligands too, such as kynurenine (the first tryptophan metabolite of the IDO pathway), was shown to optimally generate Tregs in the presence of TGF-β [39]. In our *in vitro* experiments not only did we find a conversion of naïve T cells into Tregs, but also provided support for the notion that the TCDD interfered with the primary induction of both Th1 and Th17 cells. Thus, in the presence of TCDD, TCR stimulated naïve CD4^+^ T cells cultured in conditions to cause their differentiation into either Th1 or Th17 cells, resulted in significant suppression. As it currently stands, our mechanistic experiments cannot establish which is the major explanation for the *in vivo* anti-inflammatory effects of TCDD against SK. Further investigations are needed and are underway.

The use of TCDD represents a potentially valuable approach to control SK since a single injection provided an excellent level of lesion control for at least a month pi. So far our results can only be considered as quasi therapeutic since treatment was begun 5 days after infection, a time when most infectious virus has been eliminated but clinical lesions are yet to become evident [1]–[3]. Moreover, we elected to study only the dose shown to be effective in an autoimmune disease model [9]. Since in some studies the outcome of treatment has been shown to be dose dependent, [35] similar dose response studies are warranted in the SK system and these are planned. Nevertheless, our approach does stand in contrast to most other investigations where treatment was begun prior to disease induction, or before natural disease is expected to occur. With ocular HSV infection in mice, such an early treatment approach would not be recommended because when TCDD was given one day after infection up to half of the animals succumbed to lethal infection of the CNS. Others too have observed that TCDD administration in viral infections can result in increased mortality [40]–[42]. For example, with influenza A virus infection AhR activation by the administration of TCDD decreased the survival time to lethal infection and resulted in mortality with a non lethal dose of the virus [42], [43]. The cause of lethality was unclear since TCDD treated animals cleared the virus from the lungs as well as a non treated mice [44]. More than likely animals succumbed to lung pathology associated with increased neutrophilia found in the lungs of TCDD treated mice [21], [45]. Curiously however, in our model TCDD administration reduced the numbers of neutrophils in the infected corneas.

Aesthetically, the use of TCDD for therapy, a molecule often castigated as an environmental pollutant, may have minimal appeal. However, the use of a natural ligand for AhR such as FICZ that can be metabolized by the body may not represent a good option. Several studies using FICZ have observed that inflammatory lesions can be exacerbated by such a treatment [9], [23]. For example, in the studies on EAE by Weiner and colleagues [9], FICZ treatment resulted in more severe lesions. A similar outcome was reported too by Stockinger and colleagues [23]. In our own studies, we observed no beneficial, or in fact harmful, effects when we treated HSV infected mice with FICZ. One reason AhR ligation with certain ligands can cause enhanced inflammatory lesions is that Th17 T cells, mainly responsible for mediating some inflammatory diseases, express high levels of AhR [22]17,18]. Consequently, ligation of AhR on Th17 cells can cause cell expansion and the production of cytokines that contribute to tissue damage [9], [23]. In the SK model, Th17 cells appear to play only a minor role in SK pathogenesis [46] which may explain our failure to observe adverse effects of FICZ therapy. It could be however that using non-toxic ligands such as 2-(1′H-indole-3′-carbonyl)-thiazole-4-carboxylic acid methyl ester which induces Foxp3^+^ T CD4^+^ T cells and suppresses EAE [47] could lead to a more acceptable therapeutic approach for SK.

In conclusion, our results are consistent with the observation that modulation of AhR signaling through the use of TCDD plays a role in influencing the expression of SK lesions. The mechanisms involved to explain the outcome were multiple, and involve a change in the balance between effector and regulatory T cells. We anticipate that manipulating AhR signaling, preferable with non-toxic ligands, could represent a useful approach to control an important cause of human blindness.

## Materials and Methods

### Ethics statement

This study was carried out in strict accordance with the recommendations in the Guide for the Care and Use of Laboratory Animals of the National Research Council. All animals were housed in Association for Assessment and Accreditation of Laboratory Animal Care (AAALAC)-approved animal facilities. The protocol was approved by the Institutional Animal Care and Use Committee of the University of Tennessee (PHS Assurance number A3668-01). HSV-1 eye infection was performed under anesthesia (avertin), and all efforts were made to minimize animal suffering.

### Mice, virus and cell lines

Female 6 to 8 weeks old C57BL/6 mice were purchased from Harlan Sprague Dawley (Indianapolis, IN). BALB/c DO11.10 RAG2 ^-/-^ mice were purchased from Taconic and kept in our pathogen free facility where food, water, bedding, and instruments were autoclaved. All manipulations were done in a laminar flow hood. All experiment procedures were in complete agreement with the Association for Research in Vision and Ophthalmology resolution on the use of animals in research. HSV-1 RE Tumpey and HSV-RE Hendricks were propagated and titrated on Vero cells (American Type Culture Collecting no. CCL81) using standard protocols. The virus was stored in aliquots at −80°C until use.

### Abs

CD4-allophycocyanin (RM4.5), CD4-FITC (RM4.5), Foxp3-PE (FJK-16s), anti-IFN-γ-FITC (XMG1.2), anti-IL17-PE (TC11-18H10), CD45-allophycocyanin (30-F11), CD11b-PerCP (M1/79), Ly6G-PE (1A8).

### Corneal HSV-1 infection and clinical observations

Corneal infections of C57BL/6 mice were done under deep anesthesia induced by IP injection in tribromoethanol (avertin) as previously described [48]. Mice's corneas were scarified with a 27-gauge needle, and a 3 µl drop containing the specific viral dose was applied to the eye. Eyes were examined on different days pi (dpi) with a silt-lamp biomicroscope (Kowa Company, Nagoya, Japan) measuring the progression of SK lesion severity and angiogenesis of individual mice. The scoring system was as follows: 0, normal cornea; +1, mild corneal haze; +2, moderate corneal opacity or scarring; +3, severe corneal opacity but iris visible; +4, opaque cornea and corneal ulcer; +5, corneal rupture and necrotizing keratitis [49]. The severity of angiogenesis was recorded as described previously [50]. According to this system, a grade of 4 for a given quadrant of the circle represents a centripetal growth of 1.5 mm toward the corneal center. The score of the four quadrants of the eye were then summed to derive the neovessel index (range 0–16) for each eye at a given time point.

### Treatment of animals with TCDD

TCDD (Sigma Aldrich) diluent was evaporated with nitrogen and reconstituted with DMSO. Female 6 to 8 weeks old C57BL/C mice were ocularly infected under deep anesthesia with 1×10^4^ PFU of HSV-1 RE Tumpey and divided randomly into groups. Animals in the treated groups were either treated with TCDD on day 1 pi or day 5 pi IP, being the dose administered of 1 µg/mice. Animals in the control groups were treated the same days (either day 1 or day 5 pi) with DMSO IP. Mice were observed for SK and angiogenesis progression from day 5 until day 15 or 28 as described elsewhere [49]. Most of the experiments were repeated at least three times.

### Treatment of animals with FICZ

FICZ (Biomol International, L.P., Plymouth Meeting, PA) was dissolved in DMSO. Female 6 to 8 weeks old C57BL/C mice were ocularly infected under deep anesthesia with 1×10^4^ PFU of HSV-1 RE Tumpey and divided randomly into groups. Animals in the treated groups were either treated daily with FICZ from day 1 pi to day 11 pi (IP), being the dose administered of 1 µg/mice. Animals in the control groups were treated the same days with DMSO IP. Mice were observed for HSK and angiogenesis progression from day 5 until day 15 as described elsewhere [49].

### Virus recovery and titrations

Eye swabs were taken from infected corneas using sterile swabs at the indicated time points. Infected corneas were extracted on day 6 pi and placed on ice sterile 2.0-mL straight-wall ground-glass tissue homogenizers (Wheaton) with media and homogenized. Homogenates were centrifuged (2,250 g at 4°C) for 5 min, place on ice, and immediately plated. Titrations were performed by a standard plaque assay as described previously [51]. Titers were calculated as log_10_ pfu/ml per a standard protocol [52].

### Histopathology

Eyes from control and TCDD treated mice were extirpated on day 15 pi and snap frozen in OCT compound (Miles, Elkart, IN). Six micron thick sections were cut, air dried in a desiccation box. Staining was performed with hematoxylin and eosin (Richard Allen Scientific, Kalamazoo, MI).

### Flow cytometry

#### Cell preparation

Single cell suspensions were prepared from cornea, cervical DLN, and spleen of mice at different time points pi. Corneas were excised, pooled group wise, and digested with 60 U/ml Liberase (Roche Diagnostics) for 35 minutes at 37°C in a humified atmosphere of 5% CO_2_. After incubation, the corneas were disrupted by grinding with a syringe plunger on a cell strainer and a single-cell suspension was made in complete RPMI 1640 medium.

#### Staining for flow cytometry

The single cell suspensions obtained from corneas, DLN, and spleen were stained for different cell surface molecules for FACS. All steps were performed at 4°C. A total of 1×10^6^ cells were first blocked with an unconjugated anti-CD32/CD16 mAb for 30 min in FACS buffer. After washing with FACS buffer, fluorochrome-labeled respective antibodies were added for 30 min on ice. Finally, the cells were washed three times and re-suspended in 1% *para*-formaldehyde. The stained samples were acquired with a FACS Calibur (BD Biosciences) and the data were analyzed using the FlowJo software. For corneas, total cell numbers were calculated by acquiring the totality of the sample and taking in consideration total number of corneas in the sample.

To enumerate the number of IFN-γ and IL-17 producing CD4^+^ T cells, intracellular cytokine staining was performed as previously described [5]. In brief, 10^6^ freshly isolated splenocytes, lymph node and corneal cells were cultured in U bottom 96 well plates. For *in vitro* induced cultures, cells were left unstimulated or stimulated with PMA (50 ng) and ionomycin (500ng) for 4h in the presence of brefeldin A (10 µg/ml). Subsequently, cell surface staining was performed, followed by intracellular cytokine staining using a Cytofix/Cytoperm kit (BD Pharmingen) in accordance with the manufacturer's recommendations. The Abs used were anti-IFN-γ FITC and anti-IL-17 PE. The fixed cells were resuspended in 1% paraformaldehyde. The stained samples were acquired with a FACS Calibur (BD biosciences), and the data were analyzed using the FlowJo software.

### Real time PCR

RNA was extracted from cells and tissue using TRIzol LS reagent (Invitrogen). Total cDNA was made with 500ng of RNA using oligo(dT) primer. Quantitative PCR (Q-RTPCR) was performed using SYBR Green PCR Master Mix (Applied Biosystem, Foster City, CA) with iQ5 real-time PCR detection system (Bio Rad, Hercules, CA) using 5 µl of cDNA for 40 cycles. The expression levels of different molecules were normalized to β-actin using Δ threshold cycle method calculation. Relative expression between mock infected samples and control or day 5 TCDD treated samples from day 15 pi were calculated using the 2^-ΔΔCt^ formula: ΔΔC_t_  =  ΔC_t,sample_ - ΔC_t,reference_. Here, ΔC_t_ is the change in cycling threshold between the gene of interest and the ‘housekeeping’ gene β-actin, where ΔC_t,sample_ was the C_t_ value for any day 5 TCDD treated or control samples from day 15 pi normalized to the β-actin gene and ΔC_t,reference_ was the C_t_ value for the mock infected samples (scratched and infected only with PBS) also normalized to β-actin. Each of the samples was run in duplicates to determine sample reproducibility, and a mean C_t_ value for each duplicate measurement was calculated. The PCR primers used were the following: βactin F 5′-CCTTCTTGGGTATGGAATCCTG-3′ and R 5′-GGCATAGAGGTCTTTACGGATG-3′,IL-6 F 5′-CGTGGAAATGAGAAAAGAGTTGTGC-3′ and R 5′- ATGCTTAGGCATAACGCACTAGGT-3′, TNF-α F 5′-CAGCCTCTTCTCATTCCTGCTTGTG-3′ and R 5′- CTGGAAGACTCCTCCCAGGTATAT-3′,IL-1β F 5′-GAAATGCCACCTTTTGACAG-3′ and R 5′- CAAGGCCACAGGTATTTTGT-3′,IFN-γ F 5′-GGATGCATTCATGAGTATTGC-3′ and R 5′- GCTTCCTGAGGCTGGATTC-3′,IL-17A F 5′-GCTCCAGAAGGCCCTCAG-3′ and R 5′- CTTTCCCTCCGCATTGACA-3′,IL-10 F 5′- CCTTTGACAAGCGGACTCTC-3′ and R 5′- GCCAGCATAAAAACCCTTCA-3′,CXCL-9 F 5′-CAAGCCCCAATTGCAACAAA-3′ and R 5′- TCC GGA TCT AGG CAG GTT TGA-3′,CXCL-10 F 5′-TGC TGG GTC TGA AGT GGG ACT-3′ and R 5′- AAG CTT CCC TAT GGC CCT CA-3′,CXCL-11 F 5′-GGTCACAGCCATAGCCCTG-3′ and R 5′- AGCCTTCATAGTAACAATC-3′, CCL-20 F 5′-GCCTCTCGTACATACAGACGC-3′ and R 5′- CCAGTTCTGCTTTGGATCAGC-3′.

### Purification of CD4^+^ T cells

CD4^+^ T cells were purified from pooled DLN single cell suspension obtained from HSV-infected mice using a mouse CD4^+^ T cell isolation kit (Miltenyi Biotec, Auburn, CA). The purity was achieved at the extent of 90%. Purified CD4^+^ T cells were analyzed by Flow cytometry and ELISA after stimulation for the expression of IFN-γ and IL-17.

### ELISA

DLN single cell suspensions from individual mice were collected at day 15 pi. Cells were stimulated in vitro with anti-CD3 (2 µg/ml) and anti-CD28 (1 µg/ml) for 48 h at 37°C. Additionally DLN single cell suspensions from mice were also collected at day 10 pi and CD4^+^ T cells were purified using magnetic columns. Cells were then stimulated in vitro with PMA (50 ng) and ionomycin (500 ng) for 4 h at 37°C. The concentrations of IFN-γ and IL-17 were measured by sandwich ELISA kits from eBioscience.

### In vitro induction of CD4^+^CD25^+^Foxp3^+^ regulatory T cells

Splenocytes isolated from DO11.10 RAG2 ^-/-^ mice were used as a precursor population for the induction of Foxp3^+^ in CD4^+^ T cells as described elsewhere [6]. Briefly, 2×10^6^ splenocytes after RBC lysis and several washings were cultured in 1ml volume previously optimized doses of plate bound anti-CD3 Ab (0.123 µg/ml in 200 µl total volume), rIL-2 (25–100 U/ml) and TGFβ (2.5–10 ng/ml) for 5 days at 37°C in a 5% CO_2_ incubator. Different concentrations of TCDD were also added. After 5 days samples were characterized for Foxp3 intranuclear staining using an eBioscience kit and analyzed by flow cytometry.

### Th1 and Th17 differentiation in vitro

Naïve CD4^+^ T cells were stimulated for 4 to 5 days with plate bound antibody to CD3 (4 µg/ml) and anti CD28 (2 µg/ml). For Th1 differentiation recombinant mouse IL-12 (10ng/ml) and anti IL-4 (10 µg/ml) were used. In the case of Th17 differentiation TGF-β (2.5ng/ml), IL-6 (30 ng/ml), anti IL-4 (10 µg/ml) and anti IFN-γ (10 µg/ml) were added. Concentrations of TCDD were added into cultures at the beginning of the experiment. After 5 days samples were analyzed by intracellular cytokine staining for the production of IFN-γ and IL-17 using a BD biosciences kit and then flow cytometry.

The culture mediums used were IMDM (Sigma-Aldrich) for Th17 differentiation or RPMI 1640 (Sigma-Aldrich) for Th1 differentiation, both supplemented with 2×10^−3^ M L-glutamine, 100 U/ml penicillin, 100 µg/ml streptomycin, 5×10^−5^ M β-mercaptoethanol, and 5% FCS [53].

### BrdU incorporation assay

Foxp3^+^-GFP knock-in animals were kindly provided by Dr. M. Oukka of Seattle Children's Research Institute. Mice were infected and divided into two groups: non-treated and TCDD treated mice. 8 days after ocular HSV 1 infection mice were injected IP with BrdU (1mg/mouse) and were terminated 12 hours later. 9 dpi, host Foxp3^+^CD4^+^ and Foxp3^-^CD4^+^ T cells that incorporated BrdU were analyzed by staining with anti BrdU antibody using an APC BrdU flow kit from BD Pharmingen as per the manufacturer's instructions. Samples were acquired with a FACSCalibur (BD biosciences), and the data were analyzed using the FlowJo software.

### Ex vivo apoptosis assay

DLN cells and splenocytes isolated from HSV-infected Foxp3-GFP C57BL/6 mice at 8 days pi were incubated for 5h with various concentrations of TCDD in 96 well flat-bottom plate in 5% CO_2_ incubators. After incubation period was over, cells were stained for annexin V using a kit from BD biosciences. Additionally cells were costained for CD4. Stained cells were analyzed immediately by flow cytometry.

### Statistical analysis

Most of the analyses for determining the level of significance were performed using unpaired two-tailed Student's t test. Values *P*≤0.001(***), *P*≤0.01(**), *P*≤0.05(*) were considered significant. Results are expressed as means ±SEM. For some experiments, as mentioned in the figure legends, a one-way ANOVA test was applied.

### Accession numbers for genes and proteins

CD4 (MGI:88335), IFN-γ (MGI:107656), Foxp3 (MGI:1891436), IL-17 (MGI:107364), IL-1β (MGI:96543), TNF-α (MGI:104798), IL-6 (MGI:96559), CCL20 (MGI:1329031), CXCL9 (MGI:1352449), CXCL10 (MGI:1352450), CXCL11 (MGI:1860203), IL-10 (MGI:96537), β-actin (MGI:87904), CD45 (MGI:97810), CD11b (MGI:96607), Ly6G (MGI:109440), CD3 (MGI:88332), CD28 (MGI:88327), Annexin V (MGI:106008), IL-12 (MGI:96540), IL-4 (MGI:96556), TGF-β (MGI:98725), IL-6 (MGI:96559).
